# Precarious employment at a young age and labor-market marginalization during middle-adulthood: A register-linked cohort study

**DOI:** 10.5271/sjweh.4079

**Published:** 2023-03-30

**Authors:** Emelie Thern, Nuria Matilla-Santander, Theo Bodin, Tomas Hemmingsson

**Affiliations:** 1 Institute of Environmental Medicine, Karolinska Institute, Stockholm, Sweden; 2 Department of Public Health Sciences, Stockholm University, Stockholm, Sweden; 3 Centre for Occupational and Environmental Medicine, Region Stockholm, Stockholm, Sweden

**Keywords:** disability pension, register data, sick leave, sickness absence, unemployment, young adult, young employee, young worker

## Abstract

**Objective:**

The present study aims to investigate the association between exposure to precarious employment three years after graduation and the risk of labor market marginalization (LMM) ten years later.

**Methods:**

A registered-linked cohort study based on the Swedish Work, Illness, and Labor-market Participation (SWIP) cohort was conducted among all individuals born between 1973 and 1976, who were registered in Sweden the year they turned 27 years old (N=365 702). Information on the exposure of labor market establishment three years after graduating from school and outcome of LMM ten years after graduating was collected from nationwide registers. Relative risk ratios (RRR) with 95% confidence intervals (CI) were obtained by multinominal logistic regression.

**Results:**

After considering important covariates, young adults in precarious employment three years after graduation were at an increased risk of future long-term unemployment (RRR 2.31), later precarious employment (RRR 2.85), and long-term sickness absence/disability pension (RRR 1.43) compared to individuals who had obtained standard employment arrangements within three years of graduating. Young precariously employed men had a slightly strong association compared to females with regards to all outcomes.

**Conclusion:**

The result of this study suggests that both young men and women in precarious employment three years after graduation are more likely to have a weaker attachment to the labor force later in life compared to individuals of the same age in standard employment. This is important as the prevalence of precarious employment is increasing globally, and young adults appear to be especially vulnerable.

All careers start with the first job. Some young adults establish themselves quickly, while others transition through one or several employment spells before landing the job they want. For others, securing gainful employment and a future to believe in remains a faint dream as the prevalence of permanent full-time employment is decreasing while precarious employment is increasing in Sweden and elsewhere (1–7). Precarious employment is inferior to permanent full-time employment in numerous ways and has been shown to adversely affect health (1–6). Taking a life-course perspective, the transition from education to work may be considered a difficult and sensitive period for young people (8–10). Young adults are particularly vulnerable on the labor market as they lack work experience, work opportunities, and social security benefits in case of unemployment. Furthermore, young adults are disproportionately affected by precarious employment where limited knowledge of the long-term health and work-related consequences exists (11, 12).

There has been increased recognition of the importance of conceptualizing employment status as a continuum as opposed to a dichotomy (employed and unemployed). Compared to more standard work arrangements, precarious employment is characterized by more unfavorable employment conditions, such as lack of employment contractual security (ie, temporary employment), low wage and economic deprivation, and limited social protection and workplace rights (ie, lack of benefits) (4–6). From previous research, we know that unemployment at a young age can negatively impact health and labor force participation later in life (13, 14). However, research is scarce regarding the long-term consequences of exposure to precarious employment at a young age (12, 15), which could potentially yield high societal costs.

Previous research on precarious employment at a young age has, to a great extent, relied on cross-sectional data. Results from the few studies with longitudinal data suggest an increased risk of poor mental health and worse self-rated health (15–19). Moreover, there is a gap in the literature concerning subsequent work-related outcomes such as labor market marginalization (LMM). Previous research from the field of youth unemployment has found a positive association between unemployment at a young age and later LMM (14, 20). Furthermore, precarious employment at an early stage of one’s career could potentially have a ‘scarring’ effect in terms of lower pay and lower labor force attachment, similar to exposure to youth unemployment (21, 22).

Unemployment, precarious employment, sickness absence, or receiving a disability pension can all be considered types of LMM as the individual is more distant from the labor force (23, 24). However, research within this area is scarce; one Nordic study found a positive association between precarious employment and self-reported sickness absence in the general working-age population (25).

In addition, previous research has shown that women are disproportionately affected by precarious employment (11, 26) as well as more health-related LMM (27). However, less is known about the potential differences in effects between men and women in precarious employment (2). Due to the gendered nature of the labor market and household work, it has been hypothesized that precarious employment has a greater effect on women’s health (28), which is corroborated by a previous study (29). There is, however, limited knowledge about the potential differences in effects concerning negative work-related consequences. Therefore, given that young adults and women are considered vulnerable sub-populations regarding precarious employment, it is important to further investigate the effects of precarious employment among young women.

Thus, the present study aims to investigate the association between exposure to precarious employment three years after graduation and LMM ten years later. For this purpose, we defined precarious employment using a multi-dimensional construct and used high-quality nationwide registers to follow a large cohort of individuals.

## Methods

### Study design and population

This is a register-based longitudinal study based on the Swedish Work, Illness, and Labor-market Participation (SWIP) cohort. The SWIP cohort was created by linking several nationwide registers together and includes all individuals registered in Sweden that were between 16–65 years old in 2005 and followed until 2019 (30).

The present study uses a subpopulation of the SWIP cohort, including all individuals in Sweden born between 1973 and 1976 (N=472 171). In the current study, we focused on young people and thus only included individuals who had graduated from primary, secondary, or university education before the age of 27. This age was chosen to ensure that information on the exposure status was collected when the individuals were aged ≥30 years. Consequently, individuals with missing information on education at age 27 or who continued studying after age 27 were excluded (N=104 496).

Furthermore, individuals were excluded if they were on disability pension or died before baseline (three years after graduating from school) as they were no longer at risk (N=1953). More of the excluded individuals were female, born outside of Sweden, and with worse parental socioeconomic status (SES) (supplementary material, www.sjweh.fi/article/4079, table S1). The final analytical sample consisted of 365 702 individuals. Ethical approval was obtained from the Swedish Ethical Review Authority (no. 2019-04343).

### Exposure – labor market establishment three years after graduation

Information on labor market establishment was obtained from the Longitudinal Integration Database for Health Insurance and Labour Market Studies (LISA). To minimize capturing the initial unstable labor market attachment in the immediate years after graduating from school, the exposure information of labor market establishment was obtained three years after graduation (10). Information on the year of graduation from primary (≤9 years), secondary (10–12 years), or university education (>12 years) was derived from the LISA register for the year the individuals turned 27 years old. A crude measure of the year of graduation was calculated for the individuals who had missing information on this variable (10.3% of the analytical sample). This was done by using the median year of graduation (using information from the individuals without missing information on this variable) given the level of education for each birth cohort.

The study population was categorized in the following order: precarious employment relationship (PER), long-term unemployed, sub-standard employment relationship (SSER), standard employment relationship (SER), and other, to create five mutually exclusive groups. SER served as the reference category.

PER was defined using version 2.0 of the Swedish Registered-based Operationalization of Precarious Employment (SWE-ROPE) (31). To cover the three dimensions of precarious employment identified by Kreshpaj et al (32) – employment insecurity, income inadequacy, and lack of rights and protection – the SWE-ROPE consists of five components: (i) contractual employment insecurity, (ii) temporariness, (iii) multi-job-holding, (iv) income level, and (v) coverage under collective bargaining agreement. Following the SWE-ROPE method, each individual was scored on the five components, resulting in a precarious score of -9–2 (33). Individuals were defined as being in PER if their total precarious score was <-3. A score of ≥0 was considered as SER (33, 34). The group scoring -3– -1 was more difficult to assess and was labeled as SSER (34). This distinction of SSER was established to create a distance between PER and SER. The precarious score was calculated on the employed working population only. Students, those with no registered income, and the self-employed were subsequently excluded when the precarious score was calculated for each year of LISA.

Individuals registered as unemployed for ≥180 days in one year – year three years after graduation – were defined as being long-term unemployed. Individuals not classified by any of the other groups (ie, self-employed, no registered income, not registered as students, not precariously employed with short-term employment) were defined as ‘other’. This group was created to reduce the potential issue of selection bias that could arise when excluding individuals not included in any other group.

### Outcome – labor market marginalization

Adapted from previous research, LMM was defined using four different measures: long-term unemployed, precarious employment, long-term sickness absence, and disability pension (23, 24). Long-term unemployment and precarious employment were defined in the same way as the exposure variables. Receiving sickness benefits from the Swedish Social Insurance Agency for ≥90 days annually was defined as being on long-term sickness absence. First-time, either full or partial, recipiency of disability pension was considered as having the outcome of interest. Given the few cases of disability pension and the fact that long-term sickness absence is strongly correlated to disability pension, these two outcomes were merged into one. If an individual had more than one outcome (long-term unemployment and/or precarious employment and/or long-term sickness absence/disability pension) at follow-up, then they were considered to have a combined LMM. This was done as we do not know when during the year the different outcomes occur as we only have access to annual data. Information on the outcome was obtained for one year, ten years after graduation from school (ie, seven years after the exposure of labor market establishment was measured).

### Covariates

The selection and inclusion of individual and family-level covariates were based on previous research (11, 26). Using the Multi-Generation Register (MGR), Population and Housing Censuses 1985, LISA register and Hospital Discharge Register, we extracted the following variables: sex, birth year, age at baseline, country of birth (Sweden, outside of Sweden), the highest level of education (primary, secondary, university), prior psychiatric diagnosis requiring inpatient care (ICD 10: F00-F99, and corresponding ICD-9 codes), the highest level of parental education (primary, secondary, university) and SES (non-manual, manual, self-employed/farmer and not classified). All individual and family-level covariates were categorized as indicated in table 1.

**Table 1:** Baseline characteristics of the study population (born 1973–1976), stratified by labor market establishment three years after graduation. [PER=precarious employment relation; SSER= sub-standard employment relation; SER=standard employment relation; SES=socioeconomic status; SA=sickness absence; DP=disability pension; LMM=labor market marginalization combined].

	PER		Long-term unemployed		SSER		SER		Other		P- value
					
	N (%)	Mean (SD)	N (%)	Mean (SD)	N (%)	Mean (SD)	N (%)	Mean (SD)	N (%)	Mean (SD)	
Total	45 845 (12.5)		28 877 (7.9)		113 878 (31.1)		139 598 (38.2)		37 504 (10.3)		
Sex											<0.001
Male	23 579 (51.4)		16 900 (58.2)		59 140 (51.9)		74 240 (53.2)		18 149 (48.4)		
Female	22 266 (48.6)		11 977 (41.5)		54 738 (48.1)		65 358 (46.8)		19 355 (51.6)		
Country of birth											<0.001
Sweden	40 797 (89.0)		25 066 (86.8)		105 548 (92.7)		131 677 (94.3)		27 526 (73.4)		
Outside of Sweden	5048 (11.0)		3811 (13.2)		8330 (7.3)		7921 (5.7)		9978 (26.6)		
Birth year											<0.001
1973	8491 (8.9)		9522 (9.9)		27 848 (29.0)		40 681 (42.4)		9395 (9.8)		
1974	10 888 (11.4)		8782 (9.2)		29 111 (30.4)		36 877 (38.4)		10 272 (10.7)		
1975	12 477 (14.0)		6530 (7.3)		28 347 (31.8)		32 625 (36.6)		9291 (10.4)		
1976	13 989 (16.5)		4043 (4.8)		28 572 (33.8)		29 415 (34.8)		8546 (10.1)		
Age at baseline		23.7 (3.2)		22.9 (3.3)		24.4 (3.4)		24.9 (3.5)		23 (3.6)	
Education											<0.001
Primary	3488 (7.6)		4000 (13.9)		5287 (4.6)		7030 (5.0)		8965 (23.9)		
Secondary	29 956 (65.3)		19 606 (67.9)		63 511 (55.8)		62 553 (44.8)		19 530 (52.1)		
University	12 401 (27.1)		5271 (18.3)		45 080 (39.6)		70 015 (50.2)		90009 (24.0)		
Prior psychiatric diagnosis	1369 (3.0)		1086 (3.8)		2384 (1.7)		2336 (1.7)		2476 (6.6)		<0.001
Parental education											<0.001
Primary	6346 (13.8)		5497 (19.0)		15 700 (13.8)		18 909 (13.6)		6431 (17.2)		
Secondary	21 225 (46.3)		14 544 (50.4)		54 35 (47.7)		65 179 (46.7)		14 636 (39.0)		
University	14 661 (32.0)		6435 (22.3)		37 370 (32.8)		49 053 (35.1)		9092 (24.2)		
Missing	3613 (7.9)		2401 (8.3)		6483 (5.7)		6457 (4.6)		7345 (19.6)		
Parental SES											<0.001
Non-manual	22 588 (49.3)		10 970 (38.0)		60 365 (53.0)		78 475 (56.1)		13 186 (35.2)		
Manual	15 014 (32.8)		12 142 (42.1)		37 822 (33.2)		45 672 (32.7)		11 469 (30.6)		
Self-employed/farmer	1973 (4.3)		976 (3.4)		4939 (4.3)		5102 (3.7)		1515 (4.0)		
Not classified	6270 (13.7)		4789 (16.6)		10 752 (9.4)		10 349 (7.4)		11 334 (30.2)		
Outcome											<0.001
Long-term unemployed	2520 (5.5)		2741 (9.5)		4169 (3.7)		3.654 (2.6)		3313 (8.8)		
Precarious employment	13 731 (30.0)		7588 (26.3)		25 126 (22.1)		20 489 (14.7)		9276 (24.7)		
SA/DP	509 (11.0)		4277 (14.8)		11 083 (9.7)		12 706 (9.1)		6802 (18.1)		
Combined LMM ^a^	1340 (2.9)		1166 (4.0)		2107 (1.9)		1609 (1.2)		1321 (3.5)		

a LMM having two or more of the outcomes during the same year of the follow-up.

### Statistical analysis

Pearson’s chi-square tests (χ^2^) were used to test for differences in baseline characteristics of the study population. The association between labor market establishment and later LMM was estimated by multinominal logistic regression to obtain relative risk ratios (RRR) with 95% confidence intervals (CI). The outcome was studied ten years after graduation and categorized into four different groups: long-term unemployed, precarious employment, long-term sickness absence/disability pension, and combined LMM. We used those without any LMM during follow-up as the base category and SER as the reference category.

Analyses were performed both on the total analytical sample and stratified by sex given that previous research suggests that precarious employment is more common among women, and the consequences tend to differ between men and women (18, 29). Adjustments for sex (total analyses), country of birth, birth year, age at baseline, and own highest level of education, as well as either parent’s highest level of socioeconomic status, and educational qualifications were done in the analyses.

To examine if the risk estimates differ depending on the level of education, additional analyses were performed on the total analytical sample stratified by the highest level of own education. In addition, to assess whether any elevated risk found among individuals in precarious employment was driven by short-term unemployment (<180 days), individuals reporting any form of unemployment the same years as being in precarious employment were re-categorized into the group ‘other’ in a sensitivity analysis (N=27 857). Additional sensitivity analyses were conducted excluding all the individuals with missing information on the year of examination (N=37 554). Completeness of the information on the year of examination from school depends on the source of the information, which differs both depending on the year of birth and country of birth such that certain birth cohorts and individuals born outside of Sweden had more missing on this variable. The bivariate associations between each covariate and the LMM outcomes are shown in supplementary table S2. Missing values on covariates were coded as separate categories, as similar results were obtained when individuals with missing information on covariates (N=26 299) were excluded (ie, complete case analyses) (supplementary table S3). All analyses were performed using Stata Statistical Software: release 17 (StataCorp, College Station, TX, USA).

## Results

### Baseline characteristics and prevalence of labor market marginalization states

Three years after graduating from school, most of the study population was in either SER or SSER (table 1). Around 12.5% were defined as being in PER (51.4% men, 48.6% women). Generally, the precariously employed had worse mental health, lower education and lower parental SES compared to individuals in SER but were generally better off compared to individuals in long-term unemployment.

Ten years after graduation, a total of 8429 (2.3%) individuals were on long-term unemployment, 24 870 (6.8%) individuals were precariously employed, and 15 968 (4.3%) were on long-term sickness absence or disability pension. There were 3574 (1%) individuals who experienced ≥2 of the outcomes during the same year (combined LMM).

### Associations of labor market establishment at a young age and later LMM

Table 2 shows the association between labor market establishment at a young age and later LMM for males and females combined and separately. In the fully-adjusted analyses that combined men and women, we found that being in PER three years after graduation was associated with an increased risk of being marginalized from the labor market later in life, especially long-term unemployment (RRR 2.31), precarious employment (RRR 2.85), and combined LMM (RRR 3.04) compared to individuals in SER with no LMM. Individuals defined as being long-term unemployed and SSER were also at an increased risk of later LMM ten years after graduation compared to those defined as being in SER.

**Table 2:** Crude and adjusted relative risk ratio (RRR) with 95% confidence intervals (CI) for the association between labor market establishment and later labor market marginalization ten years after graduating from school, for all and stratified by sex. [PER=precarious employment relation; SSER=sub-standard employment relation; SER=standard employment relation; SA=sickness absence; DP=disability pension; LMM=labor market marginalization; Ref=reference category].

	Long-term unemployed		Precarious employment		Long-term SA/DP		Combined LMM ^a^	
			
	Crude RRR (95%CI)	Adjusted RRR ^b^ (95%CI)	Crude RRR (95%CI)	Adjusted RRR ^b^(95%CI)	Crude RRR (95%CI)	Adjusted RRR ^b^(95%CI)	Crude RRR (95%CI)	Adjusted RRR ^b^(95%CI)
All								
PER	2.96 (2.76–3.19)	2.31 (2.14 –2.49)	3.37 (3.24–3.51)	2.85 (2.74–2.97)	1.77 (1.68–1.87)	1.43 (1.35–1.51)	3.87 (3.47–4.32)	3.04 (2.72–3.41)
Long-term unemployed	6.15 (5.74–6.60)	4.09 (3.80–4.39)	3.16 (3.01–3.31)	2.44 (2.32–2.56)	2.91 (2.75–3.08)	2.11 (1.99–2.23)	5.92 (5.28–6.63)	4.15 (3.69–4.66)
SSER	1.70 (1.59–1.82)	1.54 (1.45–1.65)	1.86 (1.79–1.93)	1.74 (1.68–1.81)	1.24 (1.19–1.30)	1.14 (1.09–1.20)	2.03 (1.83–2.25)	1.84 (1.66–2.04)
SER	Ref	Ref	Ref	Ref	Ref	Ref	Ref	Ref
Other	4.78 (4.46–5.12)	2.83 (2.63, 3.05)	3.01 (2.88–3.15)	2.16 (2.06–2.26)	4.63 (4.42–4.84)	2.89 (2.75–3.04)	5.74 (5.15–6.40)	3.53 (3.15–3.96)
Men								
PER	3.03 (2.75–3.33)	2.40 (2.18–2.65)	3.49 (3.30–3.69)	2.94 (2.77–3.11)	2.03 (1.85–2.22)	1.62 (1.47–1.78)	4.12 (3.51–4.83)	3.23 (2.75–3.81)
Long-term unemployed	6.47 (5.92–7.08)	4.52 (4.12–4.95)	3.30 (3.10–3.52)	2.55 (2.39–2.73)	3.66 (3.50–3.99)	2.52 (2.30–2.76)	6.16 (5.25–7.23)	4.39 (3.73–5.17)
SSER	1.71 (1.57–1.87)	1.55 (1.42–1.69)	1.80 (1.71–1.89)	1.67 (1.59–1.76)	1.27 (1.17–1.37)	1.15 (1.07–1.25)	1.97 (1.69–2.29)	1.77 (1.53–2.07)
SER	Ref	Ref	Ref	Ref	Ref	Ref	Ref	Ref
Other	5.06 (4.60–5.55)	3.19 (2.89–3.52)	3.29 (3.09–3.50)	2.32 (2.17–2.48)	6.89 (6.39–7.42)	4.15 (3.82–4.49)	6.07 (5.18–7.12)	3.90 (3.30–4.61)
Women								
PER	2.90 (2.60–3.24)	2.17 (1.94–2.43)	3.24 (3.06–3.43)	2.75 (2.60–2.92)	1.62 (1.52–1.74)	1.32 (1.23–1.42)	3.63 (3.12–4.24)	2.88 (2.46–3.37)
Long-term unemployed	5.51 (4.92–6.17)	3.45 (3.07–3.88)	3.04 (2.83–3.27)	2.28 (2.12–2.45)	2.72 (2.52–2.92)	1.88 (1.74–2.02)	5.84 (4.97–6.87)	3.87 (3.27–4.56)
SSER	1.69 (1.53–1.87)	1.52 (1.38–1.68)	1.91 (1.82–2.01)	1.82 (1.73–1.91)	1.22 (1.15–1.29)	1.14 (1.08–1.20)	2.07 (1.80–2.38)	1.91 (1.66–2.20)
SER	Ref	Ref	Ref	Ref	Ref	Ref	Ref	Ref
Other	4.54 (4.09–5.04)	2.47 (2.20–2.76)	2.75 (2.58–2.93)	2.05 (1.91–2.19)	3.49 (3.29–3.70)	2.33 (2.18–2.48)	5.36 (4.63–6.21)	3.30 (2.82–3.87)

a Combined LMM: having two or more of the outcomes during the same year of the follow-up.

b Adjusted RRR: adjusted for country of birth, year of birth, age at baseline, highest levels of education, prior psychiatric diagnosis, highest level of parents’ educational attainment and SES. Using multinomial logistic regression, those with no LMM were used as the base category.

When the analyses were stratified by sex, in the fully adjusted analyses we found that the association between precarious employment at a young age and later LMM appeared slightly more pronounced among males, especially in relation to later precarious employment (RRR_Men_ 2.94, RRR_Women_ 2.75) and long-term sickness absence/disability pension (RRR_Men_ 1.62, RRR_Women_ 1.32). Similar patterns were also found among the long-term unemployed and individuals in SSER.

### Additional analyses

In the analyses stratified by the highest level of education, the association between precarious employment at a young age and later long-term unemployment and precarious employment appeared to be the strongest among the highly educated group, in both the crude and adjusted models (supplementary table S4).

Sensitivity analyses were performed excluding individuals with short-term unemployment (<180 days) from the precariously employed group, which yielded similar results as in the main analyses (supplementary table S5). Furthermore, excluding individuals with missing information on the year of examination (N=49 357) and did not change the estimation substantially (supplementary table S6).

## Discussion

The results of this study suggest that being precariously employed at a young age can have negative work-related outcomes, for both genders but especially men. Compared to same-aged individuals in SER three years after graduating from school individuals in PER and unemployment had a similarly increased risk of LMM in middle adulthood, after adjusting for several important covariates.

These results further strengthen and support the current literature on the long-term consequences of precarious employment (16–19, 22). In line with the research on the long-term outcomes of youth unemployment, we found an increased risk of later LMM among young adults in precarious employment (14, 20). This strengthens the notion that youth is a particularly sensitive time where weak attachment to the labor market can have long-term detrimental labor market outcomes for individuals and society at large (8–10). Young adults are a vulnerable group and the transition from school to work is an important time in their lives. Today the transition between school and work has become comparatively longer and a more distorted path (10). Like unemployment, there appears to be some selection effects with regards to precarious employment that could explain part of the increased risk in this group (35). The prevalence of prior psychiatric diagnoses was higher among the precariously employed and unemployed compared to the individuals in standard employment. Including this covariate in the analyses had a marginal effect on the estimates, which could be due to several reasons. First, the individuals in the inpatient care registers have quite severe mental health problems and thus only capture a small proportion of the individuals with mental health problems. Second, the exposure information was collected at a time of high unemployment which could potentially influence the direction and magnitude of the impact of health selection on the associations (36, 37). Standard employment is decreasing, and more non-standard types of employment are increasing, where young adults are more often stuck in on-demand jobs (eg, occasional jobs, involuntary part-time, gig work, internships) with lower attachment to the labor force. This is of great importance as research suggests that precarious employment adversely affects health (3, 4, 38). In line with previous research on the general working population, we found a positive association between precarious employment and sickness absence, and disability pension in middle adulthood (25). Furthermore, the results of the current study suggest that precariously employed young adults have a similar risk of later labor market marginalization as individuals who were long-term unemployed. Consequently, given the changing nature of the labor market, there is an increased risk that more young adults are set on an early downhill spiral in terms of social and health conditions.

In general, employment conditions tend to be gendered, where precarious employment is most common among women (11, 26), which could potentially strengthen the existing gender inequalities in health. However, there is limited research on the differential effect of precarious employment between men and women, and they have mostly been focused on the outcome of health (18, 29). Menéndez et al (28) hypothesized a greater effect of precarious employment on women’s health due to several reasons, such as multiple forms of gender-based labor market discrimination and the gendered division of household work (ie, caring for children), which forces more women to take part-time work which in turn results in lower wages and increased dependence of their partner’s income. Furthermore, the level of precariousness can differ between men and women, whereas men have longer working hours, unpredictable schedules and are more often uncompensated for working time, and women are overrepresented in involuntary part-time employment with lower income (39). In line with previous research on precariously employed youths and self-rated health (18), we found that precariously employed men had a slightly higher risk for both health-related (sickness absence/disability pension) and non-health related LMM (precarious employment, long-term unemployment) compared to precariously employed young women. This suggests that young men compared to women are more sensitive to precarious employment, contrary to Menéndez et al’s inverse hypothesis (28).

In the additional analyses, we found that the risks of later LMM among PER was the most pronounced among the highly educated individuals. A potential reason for this finding is the differences in reference groups when the analyses were stratified by education. In the group of highly educated individuals in the SER group, the risk of becoming precariously employed is very low, in the current study only 0.5% of the individuals in SER were precariously employed after ten years (data not shown). In the low-educated group, around 4.5% of the individuals in SER were precariously employed during follow-up. Furthermore, another potential explanation is that underemployment – a dimension of precarious employment we are unable to detect using our registers – is more common among the highly-educated group in precarious employment (39).

It should also be noted that the exposure information in the current study was collected during and immediately after the economic crisis in the 1990s that hit Sweden and many other countries (40). At the beginning of the 1990s, unemployment and particularly youth unemployment increased dramatically (from 3.4% to 18.0%) (40, 41). This can be seen in the current study as many individuals, especially females, were excluded from the analytical sample as they were studying after the age of 27 years. Another consequence of this economic crisis was that inequalities in job quality resurfaced and increased the prevalence of precarious employment (7, 42). Furthermore, the lack of job security and permanent employment has been an increasing problem for several decades, a situation which was exacerbated by the COVID-19 pandemic (43). Consequently, the employment uncertainty faced by young adults in the 1990s is quite like what today’s youth are facing, which will help us gain a better understanding of the potential future challenges for today’s young adults.

Currently, the transition from school to the workforce is much longer in Sweden and elsewhere (10). Although the general transition from school to work might differ between countries depending on welfare regimes, educational systems, and labor market regulations, the results of the current study are generalizable to other contexts as precarious employment is a global phenomenon (1, 3, 29). Furthermore, young adults are one of the most vulnerable groups in precarious employment, and the results of the current study suggest that they might be as worse off as individuals in long-term unemployment in terms of later labor market marginalization. This is of great importance as the increase in non-standard work arrangements have reduced the boundaries between employment and unemployment. Consequently, there needs to be a shift in focus, both in research and policy, from the risk and consequences of being completely excluded from the labor market to the risk and consequences of being loosely integrated into the labor market.

### Strength and weakness

A large representative cohort with long-term follow-up and registered data are major strengths of the current study. The SWIP cohort is included in a comprehensive database where several nationwide registers in Sweden have been merged for the index person, as well as their parents (30). A limitation of the cohort is that it only includes individuals registered and alive in Sweden in 2005. Although the information has been backtracked to the start of the registers there could be an issue of selection as individuals who died before 2005 were not included. Using information from a separate database, this was calculated to be around 1.5% of the study base and therefore should not have affected the results.

Another strength was that we were able to include a wide range of exposure groups to better reflect the true nature of young adults’ labor force status. However, around 14% of the analytical sample did not have information on the year of graduation in the registers for several reasons. Consequently, the median graduation year was used given the individual’s birth year and the highest level of education – which could have led to exposure misclassification. Thus, a few individuals might have been categorized incorrectly during the exposure window as we did not have data on the exact year they graduated. The robustness of the findings was tested by excluding all individuals with missing information on the exam year and similar conclusions were reached.

Although there is no universal definition of precarious employment there is a general agreement that a multidimensional construct is preferred to only using one dimension (1, 26, 33, 44). The SWE-ROPE has been constructed to be highly conservative to reduce misclassification in terms of defining too many as precariously employed. Consequently, there is a low risk that we could have misclassified the individuals in precarious employment at a young age and later in life. Furthermore, although the SWE-ROPE has not been formally validated as a scale, we see that the scale captures the group in the population that has been described to be in precarious employment (33). When the SWE-ROPE was constructed, comparisons were made using a more typological approach to identify precarious employment. There was high internal consistency between the two approaches suggesting that the scale measures what it intends to measure (33). Also, previous research using the same scale has seen an increased risk of mental health problems, stroke, and all-cause mortality among the precariously employed (34, 45, 46). A limitation of the SWE-ROPE is that we are unable to capture all the dimensions of precarious employment described in the literature due to register constraints. Additional indicators that could provide a more nuanced measure of precarious employment could be the length of the contract, working part-time, or lack of social security (33). Furthermore, given its multidimensional constructs, there is less risk of misclassification error compared to the most common proxy measure of precarious employment, ie, temporary employment (44).

Including prior mental health problems requiring inpatient care is a strength due to health selection issues. A caveat of the Hospital Discharge Register is however that the registers only capture the more severe mental health problems. Due to register constraints, we were unable to include other registers, such as the Outpatient Care Register, to better capture individuals with mental health problems before baseline.

### Concluding remarks

The results of this study suggest that young men and women who are in PER three years after graduation are more likely to have a weaker attachment to the labor force ten years later compared to same-aged individuals in SER, after adjusting for educational level and parental SES. Furthermore, the results suggest that young men are more sensitive to precarious employment compared to young women. We found risks of being marginalized later in life among the precariously employed, like being long-term unemployed at a young age. So, the question prevails: Is having any job better than no job? This is of importance as the prevalence of precarious employment is increasing globally, and young adults appear to be especially vulnerable.

### Funding

This project has been funded by grants from The Swedish Research Council for Health, Working Life and Welfare (FORTE) (Dnr: 2019-00155) and The Swedish Research Council (Dnr: 2018–01917). The funders had no role or influence over study design, data collection, analysis, interpretation of the data, manuscript writing, or the decision to publish the results

### Competing interests

The authors declare no competing interests.

## Supplementary material

Supplementary material
