# Supplementary material for: Precarious employment at a young age and labor-market marginalization during middle-adulthood: A register-linked cohort study
Source: Scand J Work Environ Health. 2023 Mar 30;49(3):201–10. doi: 10.5271/sjweh.4079 (PMC10621895; doi:10.5271/sjweh.4079)
Supplement: Supplementary material [file SJWEH-49-201-S001.pdf]

Precarious employment at a young age and labor-market marginalization during middle-adulthood: A register-linked cohort study[1]

by Emelie Thern, PhD,[2] Nuria Matilla-Santander, PhD, Theo Bodin, PhD, Tomas Hemmingsson, PhD

1. *Supplementary material*
2. *Correspondence to: Emelie Thern, Unit of Occupational Medicine, Karolinska Institutet, Solnavägen 4, 113 65 Stockholm. [E-mail: emelie.thern@ki.se]*

Supplementary table 1. Baseline characteristics of the individuals included and excluded in the analytical sample

	Included n(%)	Excluded n(%)	P- value
Total	365 702 (77.5)	106 469 (22.6)	
Sex			
Male	192 008 (52.5)	48 246 (45.3)	<0.001
Female	173 694 (47.5)	58 223 (54.7)	
Country of birth			
Sweden	330 614 (90.4)	61 539 (57.8)	<0.001
Outside of Sweden	35 088 (9.6)	44 930 (42.2)	
Birth year			
1973	95 937 (26.2)	25 778 (24.2)	<0.001
1974	95 930 (26.2)	26 598 (25.0)	
1975	89 270 (24.4)	27 392 (25.7)	
1976	84 565 (23.1)	26 701 (25.1)	
Age at baseline (mean±SD)	24.3±3.5	24.7±3.7	
Education			
Primary	28 770 (7.9)	10 796 (10.1)	<0.001
Secondary	195 156 (53.4)	33 673 (31.6)	
University	141 776 (38.8)	32 042 (30.1)	
Missing	0	29 958 (28.1)	
Prior psychiatric diagnosis	9651 (2.6)	5001 (4.7)	<0.001
Parental education			

Primary	52 883 (14.5)	8 172 (7.7)	<0.001
Secondary	169 909 (46.5)	28 249 (26.5)	
University	116 611 (31.9)	28 639 (26.9)	
Missing	26 299 (7.2)	41 409 (38.9)	
Parental SES			
Non-manual	185 584 (50.8)	36 638 (36.3)	<0.001
Manual	122 119 (33.4)	18 764 (17.6)	
Self-employed/farmer	14 505 (4.0)	2194 (2.1)	
Not classified	43 494 (11.9)	46 873 (44.0)	

SES: socioeconomic status

Supplementary Table 2: Crude Relative risk ratio (RRR) with 95% confidence intervals (CI) for the bivariate association between each covariate and later labor market marginalization.

	Long-term unemployed	Precarious employment	Long-term SA/DP	Combined LMM
	Crude RRR (95%CI)	Crude RRR (95%CI)	Crude RRR (95%CI)	Crude RRR (95%CI)
Sex				
Male (ref)	1.00	1.00	1.00	1.00
Female	0.83 (0.79- 0.86)	1.13 (1.10- 1.16)	1.99 (1.93-2.06)	1.27 (1.19- 1.35)
Country of birth				
Sweden (ref)	1.00	1.00	1.00	1.00
Outside of Sweden	2.44 (2.31-2.58)	1.38 (1.32-1.43)	1.26 (1.20-1.32)	1.75 (1.59-1.92)
Birth year				
1973 (ref)	1.00	1.00	1.00	1.00
1974	0.94 (0.89-1.00)	1.01 (0.97-1.04)	1.02 (0.97-1.06)	1.01 (0.92, 1.11)
1975	0.98 (0.93-1.04)	0.97 (0.94-1.01)	0.87 (0.84-0.91)	0.98 (0.89-1.07)
1976	0.83 (0.78-0.89)	1.00 (0.97-1.04)	0.82 (0.78-0.86)	0.82 (0.78-0.86)
Education				
Primary	8.27 (7.66-8.93)	4.73 (4.53-4.99)	7.21 (6.84-7.59)	7.78 (6.99-8.71)
Secondary	3.73 (3.51-3.97)	2.37 (2.30-2.45)	2.63 (2.52-2.74)	3.25 (2.97-3.56)
University (ref)	1.00	1.00	1.00	1.00
Prior psychiatric diagnosis	1.78 (1.58, 2.00)	1.50 (1.39-1.62)	6.14 (5.82-6.49)	3.77 (3.32-4.28)
Parental education				
Primary	2.25 (2.10-2.41)	1.51 (1.45-1.57)	1.79 (1.70-1.88)	2.01 (1.81-2.23)
Secondary	1.66 (1.57-1.76)	1.28 (1.24-1.32)	1.47 (1.70-1.88)	1.66 (1.52-1.80)
University (ref)	1.00	1.00	1.00	1.00
Parental SES				
Non-manual (ref)	1.00	1.00	1.00	1.00
Manual	1.85 (1.76-1.94)	1.34 (1.30-1.38)	1.63 (1.58-1.69)	1.70 (1.58-1.83)
Self-employed/farmer	1.11 (0.97-1.26)	1.33 (1.24-1.41)	1.14 (1.04-1.25)	1.24 (1.03-1.49)
Not classified	3.11 (2.94-3.30)	1.59 (1.52-1.65)	1.83 (1.75-1.92)	2.41 (2.19-2.64)

SES: socioeconomic status, SA: sickness absence, DP: disability pension, LMM: labor market marginalization, combined LMM: having two or more of the outcomes during the same year of the follow-up

Supplementary Table 3. Complete case analyses excluding 26 299 individuals with missing information on covariates, crude and adjusted Relative risk ratio (RRR) with 95% confidence intervals (CI) for the association between labor market establishment and later labor market marginalization ten years after graduating from school.

	**Long-term unemployed**		**Precarious employment**		**Long-term SA/DP**		**Combined LMM**	
	Crude RRR (95%CI)	Adjusted RRR (95%CI)	Crude RRR (95%CI)	Adjusted RRR (95%CI)	Crude RRR (95%CI)	Adjusted RRR (95%CI)	Crude RRR (95%CI)	Adjusted RRR (95%CI)
All								
PER	2.91 (2.69- 3.14)	2.29 (2.12- 2.48)	3.33 (3.20- 3.47)	2.81 (2.69- 2.93)	1.77 (1.67- 1.87)	1.41 (1.33- 1.50)	3.91 (3.49- 4.39)	3.07 (2.74- 3.46)
Long-term unemployed	6.08 (5.65-6.54)	4.04 (3.75- 4.35)	3.14 (2.99- 3.30)	2.41 (2.29- 2.53)	2.90 (2.74- 3.07)	2.08 (1.96- 2.21)	5.96 (5.30- 6.71)	4.15 (3.68- 4.69)
SSER	1.67 (1.56-1.79)	1.52 (1.42- 1.63)	1.84 (1.78- 1.92)	1.73 (1.67- 1.80)	1.23 (1.17- 1.29)	1.13 (1.08- 1.18)	2.01 (1.81- 2.24)	1.83 (1.64- 2.03)
SER	Ref	Ref	Ref	Ref	Ref	Ref	Ref	Ref
Other	4.51 (4.17-4.87)	2.81 (2.59- 3.04)	3.12 (2.98- 3.27)	2.24 (2.13- 2.35)	5.19 (4.95- 5.44)	3.13 (2.97-3.30)	6.06 (5.40- 6.80)	3.77 (3.34- 4.25)

Adjusted RRR: adjusted for country of birth, year of birth, age at baseline, highest levels of education, prior psychiatric diagnosis, highest level of parents' educational attainment and SES

Using multinomial logistic regression, those with no LMM were used as the base category

PER: Precarious employment relation, SSER: sub-standard employment relation, SER: standard employment relation, SA: Sickness absence, DP: disability pension, LMM: labor market marginalization, combined LMM: having two or more of the outcomes during the same year of the follow-up, Ref: reference category

Supplementary Table 4. Crude and adjusted Relative risk ratio (RRR) with 95% confidence intervals (CI) for the association between labor market establishment and later labor market marginalization ten years after graduating from school, for all and stratified by level of education.

	**Long-term unemployed**		**Precarious employment**		**Long-term SA/DP**		**Combined LMM**	
	Crude RRR (95%CI)	Adjusted RRR (95%CI)	Crude RRR (95%CI)	Adjusted RRR (95%CI)	Crude RRR (95%CI)	Adjusted RRR (95%CI)	Crude RRR (95%CI)	Adjusted RRR (95%CI)
Primary education								
PER	1.14 (0.93- 1.39)	1.42 (1.16- 1.74)	2.33 (2.97- 2.62)	2.30 (2.03- 2.61)	1.67 (1.41- 1.96)	1.77 (1.49-2.10)	1.75 (1.30- 2.36)	1.77 (1.30- 2.42)
Long-term unemployed	2.17 (1.85- 2.55)	2.52 (2.13- 2.98)	2.01 (1.78- 2.27)	2.00 (1.77- 2.27)	2.26 (1.94- 2.62)	2.30 (1.96-2.68)	2.95 (2.28- 3.82)	2.86 (2.19- 3.74)
SSER	1.12 (0.94- 1.32)	1.25 (1.05- 1.48)	1.69 (1.51- 1.89)	1.68 (1.50- 1.88)	1.49 (1.29- 1.73)	1.54 (1.32-1.79)	1.66 (1.27- 2.16)	1.66 (1.26- 2.17)
SER	Ref	Ref	Ref	Ref	Ref	Ref	Ref	Ref
Other	1.52 (1.31- 1.76)	1.75 (1.49- 2.05)	1.83 (1.65- 2.03)	1.78 (1.59- 1.99)	5.17 (4.59- 5.81)	5.09 (4.48-5.77)	2.43 (1.93- 3.08)	2.29 (1.78- 2.95)
Secondary education								
PER	2.52 (2.31- 2.76)	2.32 (2.12- 2.54)	2.46 (2.34- 2.59)	2.41 (2.29- 2.54)	1.45 (1.36- 1.55)	1.33 (1.24-1.42)	3.13 (2.74- 3.59)	2.87 (2.50- 3.29)
Long-term unemployed	4.52 (4.15- 4.94)	4.26 (3.91- 4.66)	2.17 (2.05- 2.31)	2.15 (2.03- 2.28)	2.21 (2.07- 2.37)	2.17 (2.03-2.32)	4.12 (3.57- 4.75)	4.00 (3.47- 4.62)

SSER	1.60 (1.48- 1.74)	1.56 (1.43- 1.69)	1.59 (1.52- 1.66)	1.58 (1.51- 1.65)	1.08 (1.02- 1.14)	1.05 (0.99-1.11)	1.75 (1.54- 1.99)	1.69 (1.48- 1.92)
SER	Ref	Ref	Ref	Ref	Ref	Ref	Ref	Ref
Other	3.99 (3.65- 4.37)	3.26 (2.97- 3.58)	2.15 (2.02- 2.28)	2.03 (1.91- 2.16)	2.80 (2.62- 2.98)	2.31 (2.16-2.47)	4.33 (3,76- 4.99)	3.66 (3.17- 4.24)
University education								
PER	3.41 (2.83- 4.11)	3.21 (2.66- 3.87)	4.80 (4.42, 5.20)	4.71 (4.34- 5.10)	1.43 (1.25- 1.63)	1.38 (1.21-1.58)	4.78 (3.68- 6.21)	4.57 (3.51- 5.95)
Long-term unemployed	9.31 (7.77- 11.2)	8.25 (6.86- 9.93)	4.03 (3.59, 4.53)	4.08 (3.63- 4.59)	2.08 (1.77- 2.45)	2.30 (1.94-2.72)	7.10 (5.22- 9.64)	6.86 (5.03- 9.36)
SSER	1.89 (1.62- 2.19)	1.85 (1.59- 2.15)	2.16 (2.01, 2.31)	2.12 (1.98- 2.27)	1.26 (1.16- 1.38)	1.21 (1.11-1.32)	2.53 (2.03- 3.16)	2.46 (1.97- 3.07)
SER	Ref	Ref	Ref	Ref	Ref	Ref	Ref	Ref
Other	4.88 (4.05- 5.86)	3.81 (3.15- 4.62)	3.07 (2.77, 3.41)	2.92 (2.62- 3.24)	2.86 (2.55- 3.21)	2.88 (2.56-3-25)	5.91 (4.50- 7.74)	5.25 (3.87- 6.95)

Adjusted RRR: adjusted for sex, country of birth, year of birth, age at baseline, prior psychiatric diagnosis, highest level of parents' educational attainment and SES

Using multinomial logistic regression, those with no LMM were used as the base category

PER: Precarious employment relation, SSER: sub-standard employment relation, SER: standard employment relation, , SA: Sickness absence, DP: disability pension, LMM: labor market marginalization, combined LMM: having two or more of the outcomes during the same year of the follow-up, Ref: reference category

Supplementary Table 5. Crude and adjusted Relative risk ratio (RRR) with 95% confidence intervals (CI) for the association between labor market establishment and later labor market marginalization ten years after graduating from school, re-categorized individuals with any unemployment from the precarious employment group into the group 'other'

	**Long-term unemployed**		**Precarious employment**		**Long-term SA/DP**		**Combined LMM**	
	Crude RRR (95%CI)	Adjusted RRR (95%CI)	Crude RRR (95%CI)	Adjusted RRR (95%CI)	Crude RRR (95%CI)	Adjusted RRR (95%CI)	Crude RRR (95%CI)	Adjusted RRR (95%CI)
All								
PER	1.68 (1.49- 1.90)	1.49 (1.32- 1.69)	3.28 (3.11- 3.46)	3.04 (2.88- 3.21)	1.42 (1.30- 1.54)	1.26 (1.15- 1.37)	2.78 (2.36- 3.27)	2.44 (2.07- 2.87)
Long-term unemployed	6.15 (5.74- 6.60)	4.10 (3.82- 4.40)	3.16 (3.01- 3.31)	2.45 (2.33- 2.57)	2.91 (2.75- 3.08)	2.09 (1.97- 2.21)	5.92 (6.28- 6.62)	4.16 (3.70- 4.67)
SSER	1.70 (1.59- 1.82)	1.55 (1.45- 1.65)	1.86 (1.79- 1.93)	1.74 (1.68- 1.80)	1.24 (1.19- 1.30)	1.15 (1.10- 1.20)	2.03 (1.83- 2.25)	1.84 (1.66- 2.04)
SER	Ref	Ref	Ref	Ref	Ref	Ref	Ref	Ref
Other	4.37 (4.10- 4.65)	2.80 (2.63- 3.00)	3.20 (3.08- 3.32)	2.41 (2.32- 2.51)	3.48 (3.33- 3.63)	2.30 (2.19- 2.40)	5.25 (4.76- 5.79)	3.47 (3.13- 3.84)

Adjusted RRR: adjusted for country of birth, year of birth, age at baseline, highest levels of education, prior psychiatric diagnosis, highest level of parents' educational attainment and SES

Using multinomial logistic regression, those with no LMM were used as the base category

PER: Precarious employment relation, SSER: sub-standard employment relation, SER: standard employment relation, SA: Sickness absence, DP: disability pension, LMM: labor market marginalization, combined LMM: having two or more of the outcomes during the same year of the follow-up, Ref: reference category

Supplementary Table 6. Crude and adjusted Relative risk ratio (RRR) with 95% confidence intervals (CI) for the association between labor market establishment and later labor market marginalization ten years after graduating from school, excluding individuals with missing information on the year of exam (n =49 357).

	**Long-term unemployed**		**Precarious employment**		**Long-term SA/DP**		**Combined LMM**	
	Crude RRR (95%CI)	Adjusted RRR (95%CI)	Crude RRR (95%CI)	Adjusted RRR (95%CI)	Crude RRR (95%CI)	Adjusted RRR (95%CI)	Crude RRR (95%CI)	Adjusted RRR (95%CI)
All								
PER	3.01 (2.78- 3.26)	2.34 (2.16- 2.54)	3.48 (3.34- 3.63)	2.94 (2.81- 3.07)	1.70 (1.60- 1.80)	1.37 (1.29- 1.45)	3.78 (3.37- 4.28)	3.00 (2.66- 3.39)
Long-term unemployed	6.27 (5.80- 6.76)	4.23 (3.92- 4.58)	3.18 (3.02- 3.35)	2.49 (2.36- 2.62)	2.86 (2.69- 3.04)	2.17 (2.04- 2.30)	5.70 (5.04- 6.45)	4.06 (3.58- 4.61)
SSER	1.73 (1.61- 1.85)	1.55 (1.45- 1.67)	1.90 (1.83- 1.97)	1.77 (1.71- 1.84)	1.20 (1.14- 1.26)	1.10 (1.05- 1.15)	1.99 (1.79- 2.22)	1.81 (1.62- 2.02)
SER	Ref	Ref	Ref	Ref	Ref	Ref	Ref	Ref
Other	4.60 (4.24- 4.99)	3.07 (2.82- 3.34)	3.07 (2.92- 3.23)	2.33 (2.21- 2.46)	3.81 (3.61- 4.02)	2.64 (2.49- 2.79)	5.85 (5.19- 6.61)	3.95 (3.49- 4.48)

Adjusted RRR: adjusted for country of birth, year of birth, age at baseline, highest levels of education, prior psychiatric diagnosis, highest level of parents' educational attainment and SES

Using multinomial logistic regression, those with no LMM were used as the base category

PER: Precarious employment relation, SSER: sub-standard employment relation, SER: standard employment relation, SA: Sickness absence, DP: disability pension, LMM: labor market marginalization, combined LMM: having two or more of the outcomes during the same year of the follow-up, Ref: reference category
